# Diffusion tensor imaging of the human cerebellar pathways and their interplay with cerebral macrostructure

**DOI:** 10.3389/fnana.2015.00041

**Published:** 2015-04-08

**Authors:** Zafer Keser, Khader M. Hasan, Benson I. Mwangi, Arash Kamali, Fehime Eymen Ucisik-Keser, Roy F. Riascos, Nuray Yozbatiran, Gerard E. Francisco, Ponnada A. Narayana

**Affiliations:** ^1^Department of Physical Medicine and Rehabilitation and TIRR Memorial Hermann Neuro-Recovery Research Center, University of Texas Health Science Center HoustonHouston, TX, USA; ^2^Department of Diagnostic and Interventional Radiology, University of Texas Health Science Center at HoustonHouston, TX, USA; ^3^UT Center of Excellence on Mood Disorders, Department of Psychiatry and Behavioral Sciences, University of Texas Health Science CenterHouston, TX, USA; ^4^Department of Diagnostic Radiology, Division of Neuroradiology, Johns Hopkins UniversityBaltimore, MD, USA; ^5^Department of Diagnostic Radiology, Division of Diagnostic Imaging, The University of Texas MD Anderson Cancer CenterHouston, TX, USA

**Keywords:** diffusion tensor tractography, cerebellum, spinocerebellar, cortico-ponto-cerebellar, dentate-rubro-thalamo-cortical, cerebral-cerebellar volumes, cortical thickness

## Abstract

Cerebellar white matter (WM) connections to the central nervous system are classified functionally into the Spinocerebellar (SC), vestibulocerebellar (VC), and cerebrocerebellar subdivisions. The *SC* pathways project from spinal cord to cerebellum, whereas the VC pathways project from vestibular organs of the inner ear. Cerebrocerebellar connections are composed of feed forward and feedback connections between cerebrum and cerebellum including the *cortico-ponto-cerebellar (CPC)* pathways being of cortical origin and the *dentate-rubro-thalamo-cortical (DRTC)* pathway being of cerebellar origin. In this study we systematically quantified the whole cerebellar system connections using diffusion tensor magnetic resonance imaging (DT-MRI). Ten right-handed healthy subjects (7 males and 3 females, age range 20–51 years) were studied. DT-MRI data were acquired with a voxel size = 2 mm × 2 mm × 2 mm at a 3.0 Tesla clinical MRI scanner. The DT-MRI data were prepared and analyzed using anatomically-guided deterministic tractography methods to reconstruct the *SC, DRTC, fronto-ponto-cerebellar (FPC), parieto-ponto-cerebellar (PPC), temporo-ponto-cerebellar (TPC) and occipito-ponto-cerebellar (OPC)*. The DTI-attributes or the cerebellar tracts along with their cortical representation (Brodmann areas) were presented in standard Montréal Neurological Institute space. All cerebellar tract volumes were quantified and correlated with volumes of cerebral cortical, subcortical gray matter (GM), cerebral WM and cerebellar GM, and cerebellar WM. On our healthy cohort, the ratio of total cerebellar GM-to-WM was ~3.29 ± 0.24, whereas the ratio of cerebral GM-to-WM was approximately 1.10 ± 0.11. The sum of all cerebellar tract volumes is ~25.8 ± 7.3 mL, or a percentage of 1.6 ± 0.45 of the total intracranial volume (ICV).

## Introduction

The human cerebellum lies in the posterior fossa of the cranium, is connected to the brainstem via cerebellar peduncles and is separated from the cerebrum by the tentorium. Anatomically, cerebellum is divided into 3 lobes: the flocculonodular lobe, the anterior lobe and the posterior lobe. The anterior and posterior lobes can be further divided in a midline cerebellar vermis and lateral cerebellar hemispheres. The external surface of cerebellum is composed of a highly folded layer of gray matter (GM) called the cerebellar cortex.

The cerebellum receives information through input (afferent) fibers and communicates with the rest of the central nervous system through output (efferent) fibers. The main output fibers of the cerebellum—excluding projections from vestibulocerebellum to vestibular nuclei–arise from four deep cerebellar nuclei: dentate, emboliform, globose and fastigial nuclei. All input to and output from the cerebellum traverse the cerebellar peduncles. Afferent fibers enter the cerebellum mostly through the brachium pontis or middle cerebellar peduncle (MCP) which receives fibers originated in the contralateral pontine nuclei with some additional tegmental fibers; these make up mostly for the cortico-ponto-cerebellar (CPC) tract, bringing information from the cerebral cortex. The inferior cerebellar peduncles (ICPs) also known as restiform bodies connect the medulla oblongata with the cerebellum, they have mostly afferent fibers arising from the spine (posterior corticospinal tract) and from brainstem. A smaller juxtarestiform body carries efferent fibers connecting the cerebellum and the vestibular nuclei. The superior cerebellar peduncle (SCP) also named brachium conjunctivum, connects the cerebellum with the mesencephalon and brain, and it contains mostly efferent fibers, originated in the cerebellar nuclei and on afferent tract: the anterior Spinocerebellar (SC) tract (Squire et al., 2012; Gray, 2013).

**Figure 1:**
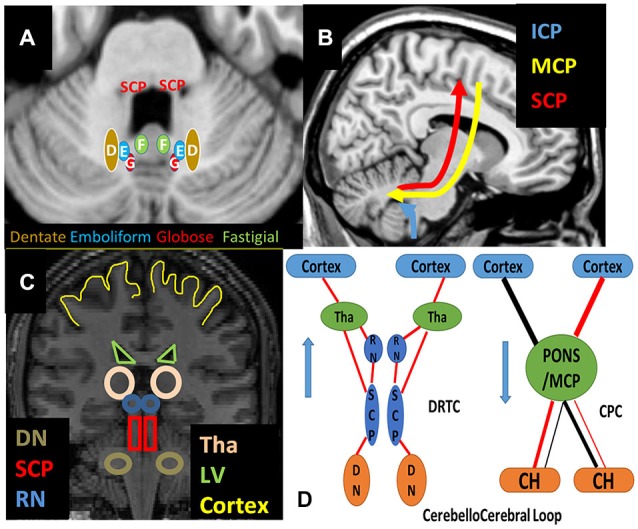
**Schematic Representation of (A) deep cerebellar nuclei on axial T1w, (B) cerebellar peduncles on sagittal, (C) relevant structures on coronal T1w and (D) cerebellocerebral loop pathways are shown. Abbreviations**: CH: Cerebellar Hemisphere, CPC: Cortico-Ponto-Cerebellar Pathways, DN: Dentate Nucleus, DRTC: Dentate-Rubro-Thalamo-Cortical Pathways, ICP: Inferior Cerebellar Peduncle, LV: Lateral Ventricles, MCP: Middle Cerebellar Peduncle, RN: Red Nucleus, SCP: Superior Cerebellar Peducles Tha: Thalamus.

The best known functional role of the cerebellum is the integration of sensory and motor functions of the brain during coordination of fine movements along with its roles in cognitive, emotional and language functions. The cerebellar connections are functionally divided into the vestibulocerebellar (VC), SC and cerebrocerebellar connections (see Figure 1; Mendoza and Foundas, 2007).The *SC* pathways project from spinal cord to cerebellum and play a role in relaying non-conscious proprioceptive/kinesthetic information from the muscle spindles and tendons, and non-conscious cutaneous feedback (e.g., pressure, touch and pain). The VC connections are the fibers originating from the vestibular organs of the inner ear and project to the brainstem and eventually to cerebellum which maintains balance and equilibrium of the body (Snell, 2009). The cerebrocerebellar connections are composed of feed-forward and feed-backward connections between cerebrum and cerebellum and considered as a loop (Habas and Cabanis, 2007a; Catani and Thiebaut de Schotten, 2012; Koziol et al., 2014). *Corticopontocerebellar (CPC)* pathways (Kamali et al., 2010; Buckner et al., 2011) of cortical origin and *dentate-rubro-thalamo-cortical (DRTC)* pathway of cerebellar origin, are responsible for fine-tuning the execution of voluntary movement and motor planning together with the high order cognitive, visual and auditory functions (Habas and Cabanis, 2007b; Blumenfeld, 2011).

Earlier clinical studies have shown that lesions in the anterior lobe and vermis of the cerebellum lead to gait and limb ataxia, dysarthria, intention tremor and hypotonia (Lee et al., 2006; Schmahmann et al., 2009). Vertigo, nausea and nystagmus are prominent in in flocco-nodular lobe injury (Duncan et al., 1975). Cognitive symptoms such as inattention, distractibility, hyperactivity, perseveration etc. (Stoodley and Schmahmann, 2009; Schmahmann, 2010; Exner et al., 2004) and behavioral symptoms such as obsessive and compulsive patterns, childishness (Riva and Giorgi, 2000) result from posterior cerebellar lesions.

Diffusion tensor imaging offers a non-invasive method to map tissue microstrutural organization by providing scalar and orientation maps that can be used to track random water molecular translational diffusion through white matter (WM) fascicles (Basser, 1997; Mori and van Zijl, 2002). Scalar maps include axial, radial, mean diffusivities and fractional anisotropy (FA). These maps are sensitive to fiber coherence in the voxel, axonal packing and myelination (Beaulieu, 2002; Hasan et al., 2011a).

Previous diffusion tensor imaging (DTI) reports have used deterministic tractography methods to study some of the cerebellar pathways in healthy subjects (Kamali et al., 2010) and patients with ataxia and cerebellar tremor (Marek et al., 2014). Probabilistic tractography methods have also been used in healthy subjects (Salamon et al., 2007), in patients with neurodegenerative diseases (Kitamura et al., 2008; Akhlaghi et al., 2014), multiple system atrophy (Shiga et al., 2005; Blain et al., 2006), pure cerebellar syndrome (Yoon et al., 2006), Parkinsonian syndromes (Nicoletti et al., 2006; Sweet et al., 2014), attention-deficit hyperactivity disorder (Ashtari et al., 2005), Asperger syndrome (Catani et al., 2008), traumatic brain injury (Spanos et al., 2007), therapy-refractory tremor (Coenen et al., 2011), blepharospasm-oromandibular dystonia (Yang et al., 2014), cerebellar mutism (Van Baarsen et al., 2013), posterior-fossa tumors (Soelva et al., 2013) and multiple sclerosis (Anderson et al., 2011). However a robust deterministic tractography protocol along with the DTI-attributes of all these pathways together with their cortical representations has not been reported.

In this study, we demonstrate the feasibility of *in vivo* delineation and 3D reconstruction of the main cerebellar pathways using high resolution DTI-based tractography in a systematic way. We have identified and quantified each cerebellar tract volume bilaterally, and presented cortical representation of cerebrocerebellar pathways in each subject native and standard space. Volume of cerebral GM, cerebral WM, cerebellar gray and WM, red and dentate nuclei (i.e., main constituents of DRTC), and the cortical thickness of the regions that cerebrocerebellar tracts are associated with, are provided to assess cerebellar and cerebral interplay in a more complete manner than attempted previously by others.

## Materials and Methods

### Subjects

This study was approved by our Institutional Review Board (IRB) and was Health Insurance Portability and Accountability Act (HIPAA) compliant. Ten right-handed healthy subjects (7 male and 3 females, age range, 20–51 years) were included in this study and written informed consent was obtained from all the subjects.

### MRI Data Acquisition

Data were acquired on a Philips 3.0 T Achieva scanner using a SENSE receive head coil. The MRI protocol included conventional MRI (dual echo turbo spin echo, phase-sensitive, Fluid attenuation by inversion recovery (FLAIR) and 3D T1-weighted magnetization prepared rapid acquisition of gradient echo (MPRAGE)). The T1-weighted sequence spatial resolution was 1 mm × 1 mm × 1 mm and field-of-view was 256 mm × 256 mm. Diffusion-weighted image (DWI) data were acquired axially from the same graphically prescribed conventional MRI volumes using a single-shot multi-slice 2-D spin-echo diffusion sensitized and fat-suppressed echo planar imaging (EPI) sequence, with the balanced and alternating polarity *Icosa21* tensor encoding scheme (Hasan and Narayana, 2003; Hasan, 2007). The b-factor = 1000 s mm^−2^, T_R_/T_E_ = 9000/65 ms, FOV = 256 mm × 256 mm and slice thickness/gap/#slices = 2 mm/0 mm/70. The EPI phase encoding used a SENSE k-space undersampling factor of two, with an effective k-space matrix of 128 × 128, and an image matrix after zero-filling of 256 × 256. The constructed image spatial resolution for the DWI data was = 1 mm × 1 mm × 2 mm. Note that the acquisition voxel volume is indeed isotropic 2 mm and this resolution is the product of upsampling.

### MRI Data Processing

The MRI data processing pipeline used in this work is described in more detail elsewhere (Hasan et al., 2011a,b; Walimuni and Hasan, 2011), and therefore we provide below a brief account of the major processing steps on both T1-weighted and DWI data.

### Tissue Segmentation and Parcellation Using T1-Weighted Data

Using FreeSurfer software library (version 5.3)^1^ (Fischl et al., 2002), the T1-weighted brain data were automatically segmented into cerebellar, brainstem and brain which included both cerebral cortex GM, sub-cortical GM, lobar WM, and cerebrospinal fluid compartments. In brief, all the T1-weighted data were visually inspected to rule out artifacts and input to FreeSurfer’s “recon-all” routine for segmentation and extraction of morphometric measurements. Specifically, processing consisted of intensity normalization, removal of non-brain tissue, Talairach transformation and segmentation of cortical and subcortical structures. FreeSurfer provided average cortical thickness and surface area using the cortical atlas labels described by Desikan et al. (2006).^2^ All volumes-of-interest were saved as labeled binary masks in each subject T1w space to enable further analyses. The total intracranial volume (ICV) was estimated by summing all segmented WM, GM and CSF from each subject. All volumes reported are adjusted for ICV (i.e., adjusted volume of region r and subject k: adjusted volume (r, k) = volume (r)/icv(k)* mean (ICV of all subjects)).

### Anatomical Landmarks of the Cerebellar Pathways

**Figure 2:**
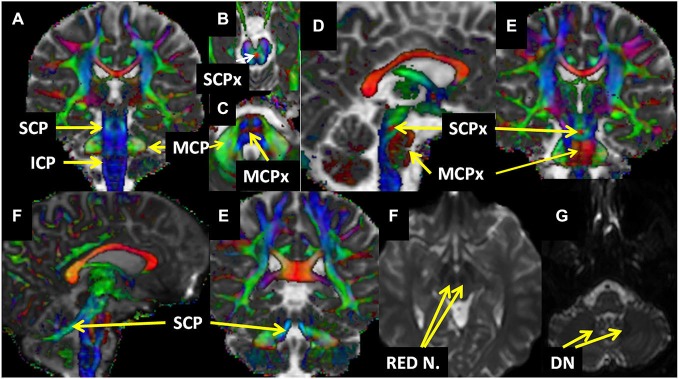
**Relevant structures who have been used in the reconstruction are presented on the fused principal eigenvector map (Red-Green-Blue) and MD (A–E) and Diffusion-weighted image (DWI) non-diffusion (F,G) maps. Abbreviations**: DN; Dentate Nucleus, ICP: Inferior Cerebellar Peduncle, MCP: Middle Cerebellar Peduncle, MCPx: Middle Cerebellar Peduncle Crossing, Red N.: Red Nuclei, SCP: Superior Cerebellar Pedunles, SCPx: Superior Cerebellar Pedunles Crossing.

**SC** pathways mainly originate from the spinal cord mainly and pass through the ICP to enter the cerebellum. **CPC** pathways arising from different parts of the cortex pass through MCP and reach to the cerebellar hemisphere. **DRTC** pathways emerging from dentate nuclei, together with interposed nuclei, traverse SCP reach to red nucleus and ventroanterior and ventrolateral nuclei (VA/VL) of thalamus then to cortex (Mendoza and Foundas, 2007; Hasan et al., 2009; Snell, 2009; Oishi et al., 2011; see Figure 2).

### Diffusion Tensor Fiber Tractography

We have used a brute force and multiple regions-of-interest (ROI) tracking method and the fiber assignment with continuous tractography (FACT) algorithm (Mori et al., 2002; Wakana et al., 2007) (DTI Studio, Johns Hopkins University, Baltimore, MD)^3^ to reconstruct *SC, DRTC, fronto-ponto-cerebellar (FPC), parieto-ponto-cerebellar (PPC), temporo-ponto-cerebellar (TPC) and occipito-ponto-cerebellar (OPC)* pathways with a FA threshold of 0.15 and an angle threshold of 70° Reproducibility of the fiber construction in both hemispheres was tested on all subjects by two experienced raters (ZK, KMH). Once a fiber tract was reconstructed, its entire trajectory was verified on a slice-by-slice basis to compare with established anatomical landmarks described in the human brain neuroanatomy atlases (Mendoza and Foundas, 2007; Snell, 2009; Blumenfeld, 2011; Keser et al., 2014).

#### Spinocerebellar Tract

**Figure 3:**
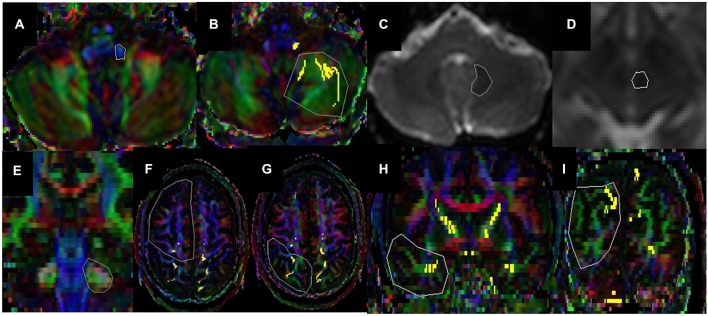
**Regions-of-interest (ROI) is seeded in (A) ICP and (B) Cerebellar Hemisphere for Spinocerebellar (SC) Tract and in (C) Dentate and (D) Red Nuclei for DRTC**. Coronal section of middle cerebellar peduncle being the first ROI for **(E)** Cortico-Ponto-Cerebellar pathways, **(F)** axial section of frontal lobe for fronto-ponto-cerebellar (FPC), **(G)** axial section of parietal lobe for parieto-ponto-cerebellar (PPC), **(H)** coronal section of temporal lobe for temporo-ponto-cerebellar (TPC) and **(I)** coronal section of occipital lobe for occipito-ponto-cerebellar (OPC) pathways are chosen as second ROI. DTI color coded (a, b and e–j and b0 (c and d) maps are used for ROI determination.

**Figure 4:**
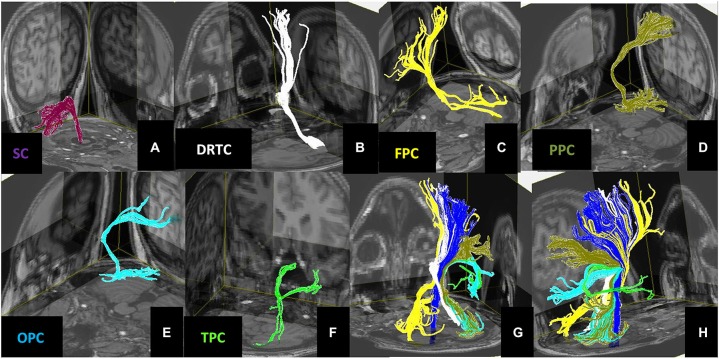
**3D views of the (A) Spinocerebellar, (B) Dentate-Rubro-Thalamo-Cortical, (C) Fronto-Ponto-Cerebellar, (D) Parieto-Ponto-Cerebellar, (E) Occipito-Ponto-Cerebellar, (F) Temporo-Ponto-Cerebellar pathways**. All these cerebellar pathways are illustrated together with corticospinal tract (dark blue) in panels **(G,H)**.

**Figure 5:**
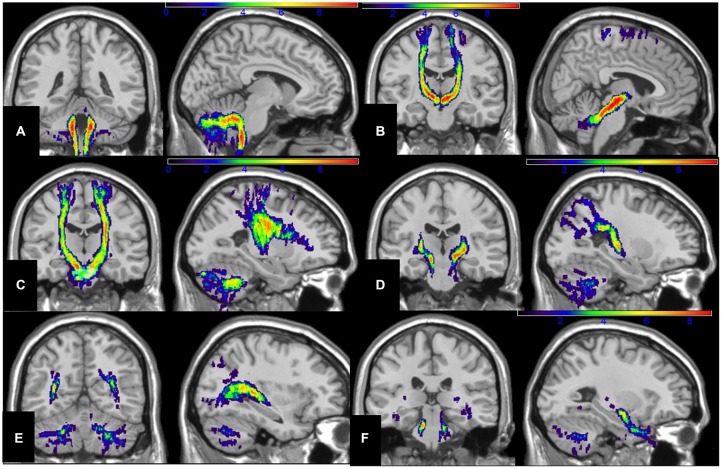
**Sagittal and coronal illustration of spatial-normalized MNI summary of the (A) spinocerebellar, (B) dentate-rubro-thalamo-cortical, (C) fronto-ponto-cerebellar, (D) parieto-ponto-cerebellar, (E) occipito-ponto-cerebellar, and (F) temporo-ponto-cerebellar are presented together with color-coded scale (blue→red; blue: 1–3 subjects, green; 4–6 subjects, yellow: 6–8 subjects, red: 8–10 subjects) that illustrates the regions of the tracts reconstructed in the number of the subjects (*n* = 10)**.

**Figure 6:**
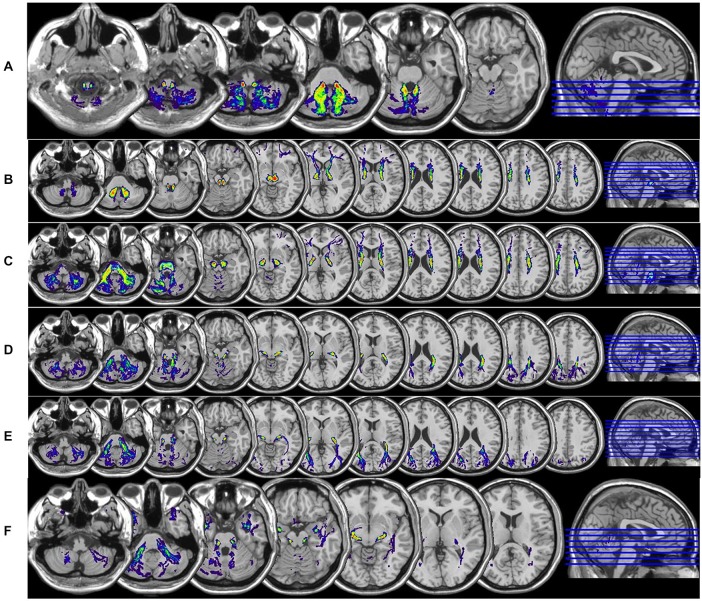
**Axial T1w sections of spatial-normalized MNI summary of (A) spinocerebellar tracts, (B) dentate-rubro-thalamo-cortical, (C) fronto-ponto-cerebellar, (D) parieto-ponto-cerebellar, (E) occipito-ponto-cerebellar and (F) temporo-ponto-cerebellar are illustrated with same scale as described in Figure 5**.

For SC (see Figures 2, 4A, 5A, 6A), first the ROI is seeded in ICP that can be seen on the axial slice of color-coded map (RGB Map) which is located at upper medulla level of the brain (see Figures 2I, 3A) and second ROI is seeded at ipsilateral cerebellar hemisphere at the same axial slice (see Figure 3B).

#### Dentate-Rubro-Thalamo-Cortical Tract

The main GM constituents of DRTC are the red and dentate nuclei have a clear contrast enhancement in the DWI non-diffusion b0 image because of iron content in adults (Hallgren and Sourander, 1958). Thus to reconstruct DRTC (see Figures 1B–D, 4B, 5B, 6B), the DWI non-diffusion b0 image was used for delineating the structures of dentate and red nuclei. The first ROI is seeded in dentate nucleus (see Figures 2F, 3D) which can be identified at the level of pontomedullary junction and second ROI in ipsilateral red nucleus (see Figures 2G, 3D) axially at the level of the cerebral peduncle in the pons.

#### Cortico-Ponto-Cerebellar Pathways

For all CPC pathways (see Figure 1D), the first ROI was placed in MCP which can be identified at the coronal section of the dorsal surface of brainstem in Red-Green-Blue color map (see Figures 2A, 3E). For FPC (see Figures 1D, 4C, 5C, 6C) the second ROI was placed in the contralateral frontal lobe which can be defined as anterior to the central sulcus at the axial level right above cingulate gyrus at the color-coded map (see Figure 3F). For PPC (see Figures 4D, 5D, 6D), the second ROI was placed in the ipsilateral parietal lobe which can be identified as the area posterior to central sulcus at the same level with FPC (see Figure 3G). For TPC (see Figures 4F, 5F, 6F), the second ROI was placed in the ipsilateral temporal lobe which can be identified at the coronal section of the anterior commissure at color-coded map (see Figure 3H) For OPC (see Figures 4E, 5E, 6E), the second ROI was placed in the ipsilateral occipital lobe at the coronal plane which is located posterior to the CC and inferior to the parieto-occipital sulcus (see Figure 3I).

### Red Nuclei and Dentate Nuclei Volumetry

Volumes of red (see Figures 2F, 3C) and dentate nuclei (see Figures 2G, 3D), which are the main constituents of the DRTC pathways, were delineated with manual ROI-based method in MRIcron software,^4^ as described elsewhere (Hasan et al., 2011a), in order to see if the right-left asymmetry present in these structures.

### Standard Space Representation of Fiber Tracts

We created a template for each DTI-derived scalar map and fiber tract using statistical parametric mapping software as the output from the DTI tractography were saved in analyze format (SPM2, Welcome Department of Cognitive Neurology, Institute of Neurology, London, UK),^5^ in MATLAB version 7.0 (The Mathworks, Inc., MA, USA). The DWI volumes with no diffusion gradient (EPI-T2-weighted images obtained with *b* = 0) of all subjects were used to build the template. Images were spatially normalized to the standard EPI template provided in SPM2 using the default parameters of SPM2 (no weighting; 25 mm cutoff; medium regularization; 16 nonlinear iterations; voxel size 1 mm × 1 mm × 1 mm; trilinear interpolation; no wrap). The normalized EPI images were visually checked for misregistration and all subjects were included in the analysis.

The fiber tracts generated in DTI studio in each subject space as binary mask was transformed into the Montreal Neurological Institute (MNI) standard brain space and a summation or visitation map was computed by repeating the procedure on all subjects. This visitation map is analogous to lesion or infarct maps (Thiebaut de Schotten et al., 2008; Hasan et al., 2011b) traditionally used to illustrate subject variability and spatial heterogeneity in standard space. In our application we have fused these tract maps with cortical and deep tissue along with their quantitative MRI (qMRI) metrics (i.e., FA, MD, and cortical thickness).

### Statistical Analyses

Group comparisons between left and right hemispheric mean values were performed using analysis-of-variance (student paired *t*-test; *F* = t*t). Correlations between regional qMRI measures were conducted using the Pearson correlation coefficient. Due to the exploratory nature of this study, we did not account for multiple comparisons and statistical significance was considered at *p* < 0.05. For the tract volumes, we also used normalized volumes of the tracts that have been scaled for each subject by the estimated ICVs (Hasan et al., 2007). Basic power analysis methods and Tables (Altman, 1980; Lachin, 1981; Van Belle and Martin, 1993; Whitley and Ball, 2002) were used to assure the utility of our work and its ability to predict sample sizes needed for future studies as has been done by others using MRI volumetry (Lange et al., 1997), cortical thickness (Han et al., 2006) or DTI-derived measurements (Wakana et al., 2007). All statistical analyses were conducted under Matlab 7.0 statistical toolbox.

## Results

We document first basic whole brain characteristics of the healthy controls used in our study. We obtained the ICV using FreeSurfer “aseg.stats”. The average total ICV of all subjects was 1622.7 ± 201.9 mL. The average ICV reported included the cerebrum, including diencephalon, brainstem, medulla oblongata and cerebellum. The total or whole brain ICV volume is composed of all ventricular and sulcal cerebrospinal fluid (CSF), deep and cortical GM, deep and lobar WM in both cerebrum and cerebellum. The qMRI and volume of left and right structures correlated strongly (*r* > 0.6; *p* < 0.01). Cerebellar pathways are reconstructed by two raters (ZK, KMH) in all subjects without any exception. Note that calculated *p*-values are uncorrected for multiple comparisons.

### Fiber Tractography and Normalized MNI Summary of the Tracts

Figure 3 illustrates the ROI placements. Figure 4 illustrates the construction of (A) SC (B) DRTC, (C) FPC, (D) PPC, (E) OPC (F) TPC and fusion with the T1w data. Spatial-normalized MNI summary of these tracts fused with T1w data is presented in Figures 5, 6.

### Cortical Representation of the Cerebellar Fiber Tracts

The cortical representation of the spatially normalized cerebellar tracts in MNI space and some general hypothesized functions of these cortical areas are summarized in Table 1. It is noteworthy that DRTC and FPC pathways have been found to be both connected with premotor cortex (Brodmann area 6).

**Table 1:** **The cortical origin and termination of the fronto-ponto-cerebellar (FPC), parieto-ponto-cerebellar (PPC), occipito-ponto-cerebellar (OPC), temporo-ponto-cerebellar (TPC), dentate-rubro-thalamo-cortical (DRTC), corticospinal (CST) and spinothalamocortical (STC) tracts are summarized**.

Name of the tract	Cortical representation (Brodmann area number)	General function of cortical areas associated with the tract (Brodmann area number)
FPC	Premotor cortex (6)	Planning of complex coordinated movements
PPC	Somatosensory association cortices (5 and 7)	Locating objects in space; where vision and proprioception converge, enabling us to determine where objects are in relation to parts of the body
OPC	Primary, secondary and associative visual cortices (17, 18 and 19)	Highly specialized for processing information about static and moving objects and is excellent in pattern recognition (17) and involved in feature-extraction, shape recognition, and visual attention (18 and 19)
TPC	Inferior temporal gyrus (20) and temporopolar area (38)	High-level visual processes and recognition (20) and important area in self representation, semantic (left) and autobiographic (right) (38)
DRTC	Premotor cortex (Area 6)	Planning of complex coordinated movements
CST	Primary motor cortex (4) and premotor cortex (6)	Execution of voluntary movements (4) and planning of complex coordinated movements (6)
STC	Primary somatosensory cortices (3, 1, and 2), Somatosensory association cortex (5) and Primary motor cortex (4)	Receiving and interpreting from the body (3, 1, 2) and locating objects in space; where vision and proprioception converge, enabling us to determine where objects are in relation to parts of the body (5 and 7)

*CST and STC are added to the table as a reference and obtained from the same ten subjects*.

### Cerebellar Tract Volumes and Corresponding DTI-Derived Average Values

**Figure 7:**
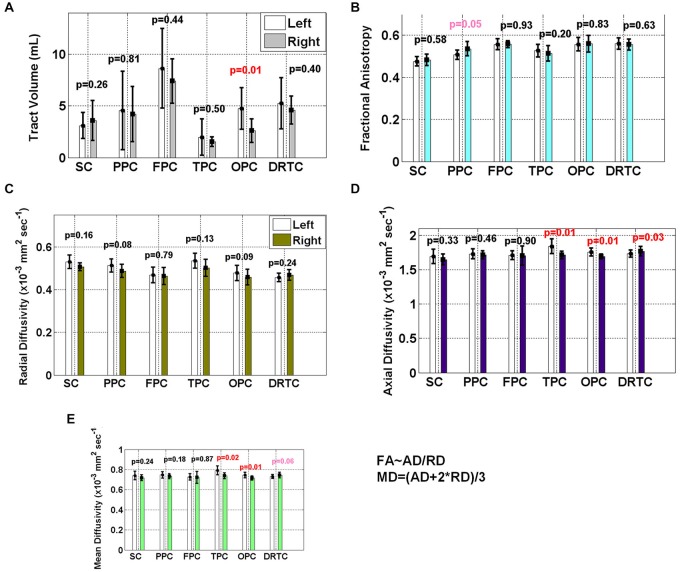
Summary of the mean and standard deviations of the cerebellar (A) tract volumes and corresponding (B) fractional anisotropy (FA), (C) radial diffusivity (RD), (D) axial diffusivity (AD) and (E) mean diffusivity (values of the bilateral SC, Dentate-Rubro-Thalamo-Cortical (DRTC), Fronto-Ponto-Cerebellar (FPC), Parieto-Ponto-Cerebellar (PPC), Temporo-Ponto-Cerebellar (TPC) and Occipito-Ponto-Cerebellar (OPC) fiber tracts on the ten subjects are provided. Note that MD = (AD + 2*RD) and FA is approximately a functional of AD-to-RD ratio (i.e., FA ~ AD/RD).

Table 2 and Figure 7 provide a summary of the mean and standard deviations of the tract volumes and corresponding DTI-derived values of the bilateral SC, DRTC, FPC, PPC, TPC and OPC on the ten healthy subjects. Note that mean and axial diffusivities of Left TPC and OPC are significantly greater than right ones (*p* < 0.05). Also Left OPC has been found to have significantly larger volume than right OPC (*p* < 0.05) and axial diffusivity of right DRTC has been significantly higher than the left hemispheric side.

**Table 2:** **Group mean and standard deviation of DTI-derived attributes of the cerebellar pathways bilaterally along with the *p* values obtained from paired *t*-test between left-right sides**.

	Tract volume (mL = cm^3^)	FA (µ ± σ)	MD (×10^−3^ mm^2^ s^−1^)	AD (×10^−3^ mm^2^ s^−1^)	RD (×10^−3^ mm^2^ s^−1^)
Left spinocerebellar	2.98 ± 1.17	0.47 ± 0.02	0.73 ± 0.04	1.15 ± 0.09	0.53 ± 0.03
Right spinocerebellar	3.40 ± 1.80	0.48 ± 0.02	0.72 ± 0.03	1.14 ± 0.07	0.51 ± 0.02
*p* value	0.26	0.41	0.58	0.33	0.25
Left DRTC	5.23 ± 2.20	0.56 ± 0.02	0.73 ± 0.02	1.28 ± 0.05	0.46 ± 0.02
Right DRTC	4.58 ± 1.21	0.55 ± 0.02	0.74 ± 0.02	1.29 ± 0.06	0.47 ± 0.02
*p* value	0.40	0.42	0.06	0.03	0.16
Left FPC	9.17 ± 3.83	0.56 ± 0.02	0.72 ± 0.03	1.24 ± 0.03	0.47 ± 0.03
Right FPC	8.07 ± 2.78	0.56 ± 0.01	0.72 ± 0.05	1.24 ± 0.10	0.46 ± 0.04
*p* value	0.44	1.00	0.74	0.90	0.68
Left PPC	4.21 ± 3.55	0.52 ± 0.03	0.74 ± 0.03	1.22 ± 0.05	0.50 ± 0.04
Right PPC	3.92 ± 2.52	0.54 ± 0.03	0.73 ± 0.02	1.23 ± 0.05	0.48 ± 0.03
*p* value	0.81	0.08	0.22	0.46	0.15
Left TPC	1.83 ± 1.62	0.53 ± 0.03	0.79 ± 0.04	1.30 ± 0.08	0.53 ± 0.03
Right TPC	1.39 ± 0.58	0.51 ± 0.03	0.74 ± 0.03	1.21 ± 0.04	0.50 ± 0.04
*p* value (Left vs. Right)	0.40	0.20	0.02	0.01	0.13
Left OPC	4.50 ± 1.93	0.55 ± 0.02	0.74 ± 0.02	1.28 ± 0.04	0.47 ± 0.02
Right OPC	2.41 ± 1.18	0.56 ± 0.04	0.71 ± 0.02	1.23 ± 0.03	0.46 ± 0.03
*p* value	0.01	0.85	0.01	0.01	0.14

Abbreviations

FA = fractional Anisotropy (no units); AD, MD and RD = axial, mean and radial diffusivities, respectively. MD = (AD + 2*RD)/3; p-value is computed from paired t-test for the comparisons of mean values between the left and right hemispheres.

*Note that the sum of cerebellar tract volumes is ~25.8 ± 7.3 mL, since ICV = 1622.7 ± 201.9 mL, therefore the total cerebellar volume-to-ICV percentage is ~1.6 ± 0.4; p (Left vs. Right) = 0.1).

### Cerebral and Cerebellar Gray and White Matter Volumetry

**Figure 8:**
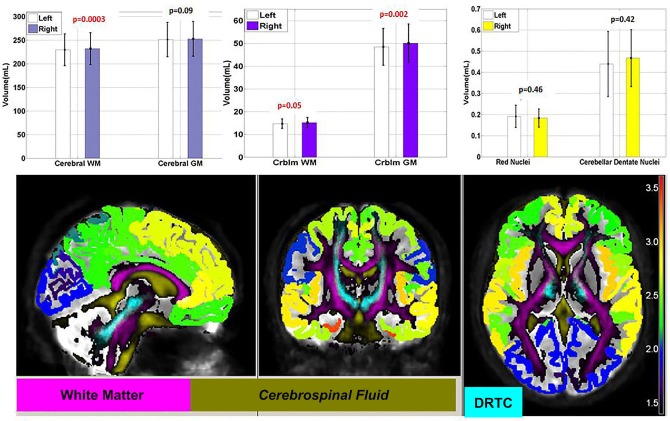
**(Upper)** Volume of cerebral and cerebellar (crblm) white (WM) and gray matter (GM) are presented. Right-Left volume asymmetry of the cerebral and cerebellar hemispheres is prominent in our ten subjects. Volumes of the red and cerebellar dentate nuclei are reported as well. **(Lower)** Results of cortical thickness of various part of right and left hemisphere illustrated with the relevant structures such as white matter (WM) (violet), cerebrospinal fluid (CSF) and dentate-rubro-thalamo-cortical tract (DRTC-light blue) The colormap of cortical thickness in mm is shown at the right side.

Volumes of cerebral gray and WM and cerebellar gray and WM are presented in Table 3 and Figure 8. In our healthy cohort, volume of right cerebellar and cerebral WM is significantly larger compared to left hemispheric WM (*p* = 0.00025). The volume of right cerebellar cortex was bigger compared to the left cerebellar cortex (*p* = 0.003). Cerebral GM-to-WM volume ratio was approximately 1.1:1 (see *Meta Analysis* in Hasan et al., 2007), whereas for the cerebellum the corresponding ratio was roughly 3.3:1. This 3-fold GM-to-WM volume ratio may offer a key clue to understanding the apparent dominance of the cerebrum over the cerebellum.

**Table 3:** **Group mean and standard deviation values of cerebral and cerebellar gray (GM) and white (WM) matter volumes, red and dentate nuclei volumes, average cortical thickness and cortical surface area of ten subjects are presented in this table**.

Quantity name (unit)	Left hemisphere	Right hemisphere	*p* value
Cerebral WM volume (mL)	229.7 ± 16.3	232.1 ± 16.3	0.0003
Cerebral GM volume (mL)	250.9 ± 12.7	252.2 ± 12.3	0.09
Cerebellar WM volume (mL)	14.7 ± 1.6	15.2 ± 1.4	0.05
Cerebellar GM volume (mL)	48.6 ± 6.8	50.2 ± 6.7	0.002
Red Nucleus volume (mL)	0.19 ± 0.03	0.18 ± 0.03	0.45
Dentate nucleus volume (mL)	0.44 ± 0.15	0.47 ± 0.12	0.42
Average global cortical	2.45 ± 0.12	2.46 ± 0.11	0.28
thickness (mm)
Cortical surface area (cm^2^)	914.6 ± 37.8	916.7 ± 38.9	0.26
Motor cortex average	2.57 ± 0.16	2.549 ± 0.178	0.28
thickness (mm)
Motor cortex volume (mL)	14.20 ± 1.21	13.99 ± 0.85	0.55

*As a reference, primary motor “precentral” cortex average thickness and motor cortex volume are included. All values are corrected for ICV except cortical thickness. p-value is computed from paired t-test for the comparisons of mean values between the left and right hemispheres*.

*Note that the total cerebellar white matter volume is slightly greater than total cerebellar tract volume (see Table 2). Cerebellar gray matter is identical to cerebellar cortex; cerebral white matter is the lobar white matter beneath the cerebral cortical sheet (see Figure 1). *The ratio of total cerebellar GM-to-WM is ~3.29 ± 0.24, whereas the ratio of cerebral GM-to WM is approximately 1.10 ± 0.11.

### Red Nuclei and Dentate Nuclei Volumetry

Volumes of red and dentate nuclei did not reveal significant right-left asymmetry (see Table 3; Figure 8) and found to be somewhat consistent with previous quantitative MRI works (Diedrichsen et al., 2011; Camlidag et al., 2014).

### Cerebellar and Cerebral Cortex Volume, Thickness and Surface Area Measurements

Bilateral cerebellar white and GM volumes and cerebral cortex volumes, surface areas, cortical thickness of lobes of the brain that CPC and DRTC pathways are associated with and average cortical surface measurement are provided in Table 3. The regional average cortical thickness values fused with DTI-derived FA and mean diffusivity is illustrated visually in Figure 8. Consistent with previous reports in healthy adults, cortical areas of occipital lobe have been found to be thinnest whereas those of temporal lobe are thickest (von Economo and Koskinas, 1925; Pakkenberg and Gundersen, 1997; Fischl et al., 2002).

### Correlation of Cortical and Subcortical Volumes vs. Cerebellar Gray and White Matter

The left Cerebellar WM volume correlated with (1) left cerebellar cortex volume *r* = 0.87 (*p* = 0.0009), (2) brainstem volume *r* = 0.68 (*p* = 0.03), (3) left hippocampus *r* = 0.74 (*p* = 0.01), (4) right cerebellar WM volume *r* = 0.91 (*p* = 0.00003), (5) right cerebellar cortex *r* = 0.86 (*p* = 0.0001), (6) right thalamus proper *r* = 0.63 (*p* = 0.05), (7) right Globus Pallidum *r* = 0.69 (*p* = 0.03), (8) right hippocampus *r* = 0.635 (*p* = 0.05). On the other hand the left and right cerebellar cortex volume correlated most strongly *r* = 0.99 (*p* = 0.0000002). Left cerebellar cortex volume correlated strongly with right cerebellar WM *r* = 0.73 (*p* = 0.02) and with right thalamus proper *r* = 0.65 (*p* = 0.04). Cerebellar white and GM did not correlate with corpus callosum volume measures. Note that all the values are corrected for ICV.

## Discussion

### Challenge of Cerebellar System Tractography and Novelty of the Work

Tracking of the cerebellar pathways is challenging because of the sharp turning angle of their course from the inferior-to-superior to right-to left orientations as these pathways cross at the level of the brainstem along with many other ascending-descending fibers such as corticospinal tracts, sensory tracts, etc. (Mori and van Zijl, 2002; Reich et al., 2006; Kamali et al., 2010). In this work, we have demonstrated that the main cerebellar pathways could be delineated and quantified using 2 mm DTI data sets. Although these tracts have been described previously, a clear deterministic tractography protocol to reconstruct all these tracts and their corresponding DTI attributes combined with their cerebral domains has not been reported.

A recent functional MRI work (Buckner et al., 2011) utilizing a large number of subjects shed light on the cortical representations of corticocerebellar connections, but did not include the cerebellar WM pathways. Our work is unique as we provided 3D reconstructions of the cerebrocerebellar tracts along with their cortical representations.

Volumetry of cerebral and cerebellar white and GM, cerebral cortex thickness and surface area measurements along with volumes of red and dentate nuclei provide a complete picture of quantification of whole system of cerebellar-cerebral connections. To the best of our knowledge, there are no previous quantitative MRI studies revealing this system in a very detailed and systematic manner as we attempted.

### Volumetric Analysis

As previously pointed out by Wakana et al. (2007), we observed a significant amount of variance in the tract volume whereas FA, MD and RD values were somewhat consistent. Even after normalization of the volumes by the estimated ICVs using both DWI-b0 and masked T1w maps (Hasan et al., 2011b); this significant variation in tract sizes was present in our cohort.

Volumetry of cerebral and cerebellar white and GM, dentate and red nuclei and computational cortical thickness measurement are reported in order to provide more concrete basis for reporting tract volumetry. Although right side of brain and cerebellum are larger in volume, the volume of most of the tracts in left side has been found to be slightly bigger in our subjects (see Figure 7). We are quite aware that differences in volumes, although significant, do not help us reach definite answer because of large variation and small differences, studies with larger sample size are needed to obtain statistically accurate answers based on power analyses (Altman, 1980; Van Belle and Martin, 1993).

### Cerebral and Cerebellar Gray Matter-to-White Matter Ratio

Our results indicate that the GM to WM ratio is strikingly higher in the cerebellum compared to cerebrum, which is consistent with previous *in vivo* MRI (Walhovd et al., 2011) and postmortem studies (Andersen et al., 2003; Azevedo et al., 2009) and comparative studies (Herculano-Houzel, 2010). The cerebellum is indeed unique despite that it occupies ~10% of the total brain volume. The cerebellar cortex (Andersen et al., 2003) is thinner than cerebral cortex (Pakkenberg and Gundersen, 1997). The cerebellar GM-to-WM volume ratio may explain why the cerebellar neurons make approximately 80% of the whole brain neurons which could have also led to cerebellum superiority in short range communication (Herculano-Houzel, 2010), in contradistinction to the cerebral cortex which controls long range top-down communications. Also cerebellar cortex gyrification is obviously greater and more complicated due to space restrictions. Also the folia structures of the cerebellum seem to have complexity far superior to the cortex. This may imply that the packing density of neurons in cerebellum is very high (Azevedo et al., 2009; Herculano-Houzel, 2010) and that cerebral cortex includes more of other non-neuronal cells such as glia.

### Right-Left Asymmetry of DTI Attributes of the Cerebellar Tracts

Asymmetry in right-left of diffusivity (MD and AD) values of TPC, OPC and DRTC together with tract volumes of OPC is somewhat unexpected. More interestingly these are the tracts that have been found to connect cerebrum and cerebellum in ipsilateral fashion. The sample size calculation has pointed out that the sample size needed to reveal statistical difference in MD values of right and left TPC and OPC along with volumes right-left OPC has been somewhat enough powerful (Altman, 1980).

### Laterality of the Cerebellar Tracts

Another aspect of our work is the laterality of the cerebellar tracts. In the literature, major part of cerebrocerebellar connections is known to cross at the pons level whereas minor part goes uncrossed. We observed that FPC which is one of the main constituent of CPC, crossed at pons level, whereas, TPC and OPC pathways which are the minor constituent of the CPC did not. This observation is somewhat consistent with the previous human neuroanatomical works (Mendoza and Foundas, 2007; Snell, 2009; Blumenfeld, 2011). However; it was surprising to see ipsilateral connection between parietal lobe and cerebellum, and dentate nucleus and red nucleus (Cicirata et al., 2005). This apparent contradiction brings up the important question that has been repeatedly asked; “how real are the *in vivo* DTI-based tractography results?” Although post-mortem human studies are the gold standard for human anatomy, we cannot underestimate consistent findings in human neuroimaging studies even though they are somewhat contradictory to the post-mortem studies. The reason why we are not seeing the crossing might be attributed to partial volume averaging due to large voxel size but our observation of ipsilateral connections cannot be underestimated either even though previous post-mortem anatomical works do not report it clearly. SC pathways are known to provide ipsilateral connection (Mendoza and Foundas, 2007; Snell, 2009; Blumenfeld, 2011) between cerebrum and cerebellum and which was consistent with our work as well.

### The Contribution of the Study Towards Understanding Cerebellar Functions

While the cerebellum’s role in motor function is well-documented, the nature of its concurrent role in high-order cognitive and emotional functions has received increased interest recently (Lee et al., 2006; Schmahmann, 2010; Koziol et al., 2014). We believe that revealing the connections of the cerebellum with extracerebellar structures is a crucial step forward in understanding the roles of human cerebellum in motor, cognitive and emotional functions. In order to highlight the potential functional roles of the cerebrocerebellar fiber tracts, we provided a summary in Table 1 that also included the corticospinal and sensory fiber tracts as a reference, despite that these tracts evolved developmentally to their final form at different fetal stages (Scott et al., 2012; Takahashi et al., 2014). Our finding that both FPC and DRTC project to similar cerebral domains provides some evidence for the presence of cerebrocerebellar loop as described by other researchers (Catani and Thiebaut de Schotten, 2012; Koziol et al., 2014; see Figure 1). Contrary to Buckner et al. (2011) report based on functional MRI, we did not observe direct connection between the FPC or DRTC pathways with Brodmann areas 9 and 46. This observation may hint that the connection between cerebellum and these prefrontal areas is through indirect pathways such as intracortical connections. Termination of PPC unto somatosensory association cortices, along with TPC and OPC projection unto higher language/visual association cortices is also one of our findings that suggest potential involvement of cerebellar pathways in other functions than motor planning and learning (Catani and Thiebaut de Schotten, 2012; Koziol et al., 2014).

### Limitations

This study is limited as the number of healthy subjects was small to test confounders such as gender or age and even hemispheric asymmetry. We provided sufficient details to help advance future experimental designs. We also did not acquire functional data on these subjects and did not include a patient group with clinical data. We acknowledge that our findings would not survive correction for multiple comparisons due to the small sample size (*n* = 10). The results should be interpreted with caution and that replications will be necessary in future works. Extensions of this work will be the enhancement in spatial and angular resolutions with bigger sample size to reduce partial volume averaging artifacts (Alexander et al., 2001), to reveal finer microstructure of the cerebellum (i.e., intra-cerebral connectivity, cerebellar thickness, cerebellar gyrification or surface folding), and to address the issue of multiple comparison. We also paved the way to future applications of the methods to patient populations such as autism, major depression, bipolar disorders, stroke, traumatic brain injury, spinal cord injury and multiple sclerosis to better reveal the role of cerebello-cortical connections and interplay of qMRI metrics with respect to clinical symptoms and potential role in recovery due to neurorehabilitation, exercise and training.

## Conflict of Interest Statement

The authors declare that the research was conducted in the absence of any commercial or financial relationships that could be construed as a potential conflict of interest.

## Footnotes

^1^http://surfer.nmr.mgh.harvard.edu/

^2^http://www.freesurfer.net/fswiki/CorticalParcellation

^3^http://cmrm.med.jhmi.edu

^4^http://www.mccauslandcenter.sc.edu/mricro/mricron/

^5^http://www.fil.ion.ucl.ac.uk/spm/
